# Comments on the Study “The Sensitivity, Specificity and Accuracy of Warning Signs in Predicting Severe Dengue, the Severe Dengue Prevalence and Its Associated Factors”

**DOI:** 10.3390/ijerph16060922

**Published:** 2019-03-14

**Authors:** Giordana Poletti-Jabbour, Natalia Elejalde-Farfán

**Affiliations:** Universidad Peruana de Ciencias Aplicadas, Av. Alameda San Marcos 360, Chorrillos, Lima 15023, Peru; nelejalde27@gmail.com

**Keywords:** dengue, severe dengue, warning signs, ns1 protein, cross-sectional studies

## Abstract

Comments were made on the article “The Sensitivity, Specificity and Accuracy of Warning Signs in Predicting Severe Dengue, the Severe Dengue Prevalence and Its Associated Factors” found in the journal “International Journal of Environmental Research and Public Health” based on the CASP Checklist’s guide for the assessment of diagnostic tests.

To the Editor,

We have reviewed with great interest the article published by Mohd Hanief Ahmad, et al. in the "International Journal of Environmental Research and Public Health", in which you are the editor [1]. The study attempts to find which warning signs and other risk factors could be associated with severe dengue. The findings of this article could help diagnose and treat dengue disease more quickly and effectively, not only by health personnel but also by family members. These findings are highly important as dengue is a very common viral infection in urban and semi-urban areas of countries with tropical climates. According to the WHO, this disease produces significant mortality, especially in children. Moreover, we know that over the last decades, its incidence has increased enormously; there are currently 390 million dengue infections per year, of which only 96 million present clinical manifestations [2].

Nevertheless, when reviewing the study using the CASP Checklist’s guide for diagnostic studies [3], we found certain aspects that could affect the interpretation of this study. Regarding the confirmatory diagnosis of dengue, the NS1 rapid test or serology were used (although neither the antibody nor the methodology used is specified). Even though, NS1 helps detect primary infections, in secondary infections it loses sensitivity, which is why it would not be the most appropriate test in areas with a high prevalence of dengue and recurrent infections [4]. In the same way, using a single serology could indicate previous infections, and not necessarily be a diagnostic test. If it had been available, the ideal would have been to use only the cases confirmed by RT-PCR, IgM seroconversion, or a 4-fold increase in IgG titers [5]. It is important to remember that diagnosis by laboratory tests must be accompanied by clinical diagnosis, especially in areas where there is coinfection with other diseases with similar clinical presentations, such as leptospirosis.

We are also concerned that records of 2607 patients who met the definition of confirmed dengue cases were not used in the analysis; instead, a sample of only 700 patients was chosen. Since a sample size or power calculation is not included, we do not know if this number was adequate to find significant differences in the subsequent analysis. Even if the sample was chosen randomly and was proportional to the months of the year, perhaps it could be.

We could also appreciate that, in the data analysis of the mentioned study, odds ratios (ORs) were used to evaluate the association with the independent variables. This measure of association can be used in cross-sectional studies as long as the prevalence of the outcome variable is low; some authors suggest 10% or less. In this case, with the prevalence of severe dengue being 4.9%, we believe that the use of ORs does not excessively affect the estimation of the risk made [6]. Concerning the calculation of sensitivity, specificity, positive and negative predictive values, we congratulate the authors for including an explanatory table; however, the parameters next to the confidence intervals must not be forgotten in order to assess the accuracy of the estimates. Furthermore, reporting the positive and negative likelihood ratios is always very useful information for studies on diagnostic tests.

Finally, having a robust study is especially important when the results reported disagree with what has already been published in the scientific literature. For example, the authors found no association between severe dengue with age and comorbidities, factors widely described in the literature [7]. We believe that the use of an adequate test to define the participants, a better explanation of the selection of the sample, and including a history of previous infection could have strengthened the study enormously, giving greater certainty to the results found.
